# Supplementary material for: Plasma lipidome profiling of newborns with antenatal exposure to Zika virus
Source: PLoS Negl Trop Dis. 2021 Apr 30;15(4):e0009388. doi: 10.1371/journal.pntd.0009388 (PMC8115770; doi:10.1371/journal.pntd.0009388)
Supplement: S1 Table — (DOCX) [file pntd.0009388.s004.docx]

**S1 Table. Epidemiological description of samples.**

| **Group** | **ID** | **Intergrowth classification** | **NB ZIKV** | **Sex** | **Birth** | **Head circumference (cm)** | **Predicted maternal comorbidity** | **Complication during pregnancy** | **Types of birth** | **Maternal anti-zika IgG** | **Maternal rash** | **Rash gestational trimester** | **NB Hb (g/dL)** | **NB Hct (%)** | **NB WBCs (cells/µL)** |
| --- | --- | --- | --- | --- | --- | --- | --- | --- | --- | --- | --- | --- | --- | --- | --- |
| G1 | JM10 | Normocephalic | N | M | 2016-02-21 | 33 | No | HBP | Vaginal | N | Yes | NI | NI | NI | NI |
|  | JM104 | Normocephalic | N | M | 2016-03-06 | 34.5 | No | No | Vaginal | N | Yes | First | 17.1 | 45.2 | 8200 |
|  | JM108 | Normocephalic | N | F | 2016-03-05 | 32 | No | No | Vaginal | N | No | NI | NI | NI | NI |
|  | JM109 | Normocephalic | N | M | 2016-03-07 | 32 | No | No | Vaginal | N | No | NI | 19.9 | 49.7 | 9900 |
|  | JM112 | Normocephalic | N | M | 2016-03-09 | 32 | No | No | Vaginal | N | No | NI | 13.5 | 35.5 | 15800 |
|  | JM140 | Normocephalic | N | M | 2016-04-02 | 34.5 | No | No | Vaginal | N | Yes | First | NI | NI | NI |
|  | JM150 | Normocephalic | N | M | 2016-04-06 | 34 | No | No | Vaginal | N | Yes | Third | 15.5 | 41.5 | 10700 |
|  | JM183 | Normocephalic | N | F | 2016-05-28 | 34 | No | No | Vaginal | N | No | NI | NI | NI | NI |
|  | JM29 | Normocephalic | N | M | 2016-02-02 | 34 | No | No | Cesarian | N | Yes | First | 11.9 | 31.5 | 9000 |
|  | JM167 | Normocephalic | N | F | 2016-05-03 | 32 | No | No | Vaginal | N | Yes | First | NI | NI | NI |
| G2 | JM02 | Normocephalic | P | F | 2016-02-20 | 32.5 | No | No | Vaginal | P | Yes | First | NI | NI | NI |
|  | JM06 | Normocephalic | P | M | 2016-01-20 | 33 | No | No | Vaginal | P | Yes | Second | 18 | 49.2 | 14300 |
|  | JM120 | Normocephalic | P | F | 2016-03-14 | 29 | No | No | Cesarian | P | Yes | NI | 16.0 | 43.6 | 21500 |
|  | JM134 | Normocephalic | P | F | 2016-03-23 | 37 | No | No | Cesarian | P | Yes | First | NI | NI | NI |
|  | JM28 | Normocephalic | P | M | 2016-02-01 | 37 | HBP | HBP | Vaginal | P | Yes | First | NI | NI | NI |
|  | JM54 | Normocephalic | P | M | 2016-01-31 | 34.5 | No | No | Vaginal | P | No | NI | 11.7 | 30.7 | 9600 |
|  | JM56 | Normocephalic | P | M | 2016-02-15 | 35.6 | No | No | Vaginal | P | Yes | First | NI | NI | NI |
|  | JM75 | Normocephalic | P | F | 2016-02-22 | 34 | No | No | Cesarian | P | Yes | First | 11.1 | 34.3 | NI |
|  | JM77 | Normocephalic | P | M | 2016-02-20 | 33 | HBP | No | Cesarian | P | Yes | Third | 22 | 58 | 10000 |
| G3 | JM119 | Severe microcephalic | P | F | 2016-03-16 | 25.5 | No | No | Vaginal | P | Yes | NI | 20.9 | 53.4 | 7000 |
|  | JM145 | Microcephalic | P | F | 2016-04-06 | 31 | HBP | HBP | Vaginal | P | No | NI | 21.4 | 56.3 | 15400 |
|  | JM38 | Microcephalic | P | M | 2016-02-09 | 31 | No | No | Vaginal | P | No | NI | NI | NI | NI |
|  | JM72 | Microcephalic | P | M | 2016-02-20 | 32 | No | No | Vaginal | P | No | NI | 19.9 | 53.8 | 11600 |
|  | JM01 | Severe microcephalic | P | M | 2016-01-17 | 27 | No | No | Vaginal | P | No | NI | 18.2 | 47.3 | 14800 |
|  | JM171 | Microcephalic | P | F | 2016-05-08 | 31 | ET | No | Cesarian | P | No | NI | NI | NI | NI |
|  | JM178 | Microcephalic | P | F | 2016-05-12 | 30 | HBP | PE | Vaginal | P | No | NI | 17.8 | 47.4 | 11800 |
|  | JM192 | Microcephalic | P | F | 2016-09-18 | 31 | No | No | Vaginal | P | Yes | NI | 20.4 | 54.2 | 19400 |
|  | JM198 | Microcephalic | P | F | 2016-10-24 | 31 | No | No | Vaginal | P | Yes | Third | 19.3 | 51.8 | 22400 |
|  | JM200 | Severe microcephalic | P | F | 2016-11-30 | 27 | No | No | Vaginal | P | Yes | NI | NI | NI | NI |
|  | 6905/17 | Microcephalic | P | M | 2017-07-10 | 30.5 | NI | NI | NI | NI | NI | NI | NI | NI | NI |
| ID: Identification; F: Female; M: Male; NB: Newborn; P: Positive; N: Negative; NI: No information; HBP: High blood pressure; ET: Essential thrombocythemia; PE: pre-eclampsia Hb: Hemoglobin; Hct: Hematocrit; WBCs: White blood cells. | | | | | | | | | | | | | | | |
