# Supplementary material for: Plasma lipidome profiling of newborns with antenatal exposure to Zika virus
Source: PLoS Negl Trop Dis. 2021 Apr 30;15(4):e0009388. doi: 10.1371/journal.pntd.0009388 (PMC8115770; doi:10.1371/journal.pntd.0009388)
Supplement: S1 Methods — (DOCX) [file pntd.0009388.s007.docx]

**S1 Methods.** **Detailed description of methods applied to plasma lipidome profiling**

Umbilical cord blood samples were obtained in EDTA tubes, kept on ice before centrifugation at 1,200 x *g* for 15 min at room temperature. Aliquots of plasma samples were stored at -80 °C until analyses. Plasma lipidome extraction was performed with 20 µL of plasma samples, a pooled aliquot of all samples (quality controls) or ultrapure water (extraction blanks), spiked with 50 μL of an internal standard mixture (Table 1) and the volume adjusted to 450 μL using ice-cold methanol. One mL of methyl tert-butyl ether (MTBE) was added to this solution, thoroughly vortexed for 15 s and stirred for 1h at 20 ºC. After centrifugation at 10,000 x *g* for 10 min at 4 °C, the supernatant was transferred to a glass vial, dried under N_2_ gas and dissolved in 100 μL of isopropanol. The lipid extraction protocol followed the methods by [1].

The lipid extracts were analyzed by ultra high-performance liquid chromatography (UHPLC Nexera, Shimadzu, Kyoto, Japan) tandem electrospray ionization time-of-flight mass spectrometry (ESI-Q-TOFMS, Triple TOF 6600, Sciex, Concord, US). Here we applied an untargeted lipidomic approach developed by our group [2,3] using reverse-phase LC-MS in both positive and negative ionization modes. The lipid extracts of experimental samples were randomized and injected (1 µL injection volume) in batches of 5 samples, and a blank of injection (isopropanol), an extraction blanks and a quality control sample were analyzed at the beginning and end of each batch. Samples were loaded into a CORTECS (UPLC C18 column, 1.6 µm, 2.1 mm i.d. x 100 mm) with a flow rate of 0.2 mL/min and the oven temperature maintained at 35 °C. The mobile phase A consisted of water/acetonitrile (60:40), while mobile phase B of isopropanol/acetonitrile/water (88:10:2). For experiments performed in negative or positive ionization mode, ammonium acetate or ammonium formate (at final concentrations of 10 mM), respectively, were added to mobile phases A and B. Separation of lipids was achieved by a 20 min linear gradient: from 40 to 100% B over the first 10 min., hold at 100% B from 10–12 min., decreased from 100 to 40% B during 12–13 min., and hold at 40% B until 20 min. Using Analyst 1.7.1, MS data acquisition was performed with a scanning range of 200–2000 Da, a cycle time period of 1.05 s with 100 ms for precursor ion (MS1) scan and 25 ms acquisition time to obtain the MS/MS from the top 36 precursor ions. Ion spray voltages of −4.5 kV and 5.5 kV were applied for negative and positive ionization modes, respectively, and the cone voltage set at −/+80 V. The curtain gas was set to 25 psi, nebulizer and heater gases to 45 psi and interface heater at 450 °C.

Data from MS/MS obtained by Information Dependent Acquisition (IDA) were analyzed in PeakView. Identification of lipid molecular species was exclusively based on their exact masses coupled to specific MS/MS fragments and/or neutral losses (Han, 2016). The top 400 and 300 most abundant ions in IDA were used for MS/MS identification in negative and positive ionization modes, respectively. Given the low abundance of fragment ions in MS/MS experiments, free fatty acids and free cholesterol were exceptionally identified by exact masses and retention time. Two samples per group were analyzed in each ionization mode yielding 4,200 inspected MS/MS and a total of 274 species for quantification (Table 1). Glycerophospholipids, sphingolipids and free fatty acids were identified and quantified in negative ionization mode, whereas cholesteryl esters, free cholesterol, triglycerides, acylcarnitines and coenzyme Q10 in positive ionization mode (see details in S3 Table).

Using MultiQuant lipid molecular species were quantified by comparison of chromatographic peaks of precursor ions as area ratios to those of the corresponding internal standard (Table 1) within a maximum of 5 mDa limit for attribution. Integration of chromatographic peaks was carefully performed using manual inspections of peak detection and accurate area determination. The concentration of lipid molecular species was calculated by either multiplying the area ratio by the concentration of the corresponding internal standard or by external calibration curves (PI relative to PC). The latter calculation was based on a curve containing 5 concentration points comparing PC (17:0/17:0) with PI (14:1/17:0) (0.02 to 10 ng). Internal standards for some lipid classes were unavailable in the laboratory (mono- and di-hexosyl ceramides, coenzyme Q10, free cholesterol and acylcarnitines) and quantification was based on non-specific surrogates (Table 1). Thus, the absolute concentrations for the latter compounds are not comparable to other compounds, but still largely comparable among samples.

Lastly, the reader is encouraged to contact the corresponding author for any further details on the lipid analysis, including information on MS/MS spectra and MS raw data.

**References**

1. Matyash V, Liebisch G, Kurzchalia TV, Shevchenko A, Schwudke D (2008) Lipid extraction by methyl-tert-butyl ether for high-throughput lipidomics. J Lipid Res 49: 1137–1146.

2. Chaves-Filho AB, Pinto IFD, Dantas LS, Xavier AM, Inague A, Faria RL, et al. (2019) Alterations in lipid metabolism of spinal cord linked to amyotrophic lateral sclerosis. Sci Rep 9: 11642.

3. Queiroz A, Pinto IFD, Lima M, Giovanetti M, de Jesus JG, Xavier J, et al. (2019) Lipidomic analysis reveals serum alteration of plasmalogens in patients infected with ZIKA virus. Front Microbiol 10: 753.
