# Supplementary material for: Plasma lipidome profiling of newborns with antenatal exposure to Zika virus
Source: PLoS Negl Trop Dis. 2021 Apr 30;15(4):e0009388. doi: 10.1371/journal.pntd.0009388 (PMC8115770; doi:10.1371/journal.pntd.0009388)
Supplement: S2 Methods — (DOCX) [file pntd.0009388.s008.docx]

**S2 Methods. Targeted oxilipidomic analysis of HODE and HETE**

Mono-hydroxides derived from linoleic (HODE) and arachidonic (HETE) acids were analyzed as described by [48] with some modifications. Briefly, plasma total lipid extracts were spiked with 2.5 ng of external standards (13-HODE-d4 and 5-HETE-d8). Oxidized lipids were analyzed using the same UPLC-MS/MS system described above. The samples were loaded into a UPLC BEH shield RP18 column (2.1 x 100 mm; 1.7 μm; Waters) with a flow rate of 0.5 mL/min and the oven temperature maintained at 35 °C. The mobile phase A consisted of acetic acid/water/acetonitrile (0.02:50:50), while mobile phase B composed of acetic acid/acetonitrile/isopropanol (0.02:50:50). Oxidized lipids were separated by a 10 min linear gradient as follows: from 0.1 to 55% B over the first 4 min., from 55 to 99% B from 4.0-4.5 min., hold at 100% B from 4.5-6.5 min., decreased from 99 to 0.1% B during 6.5–7 min., and hold at 0.1% B from 7–10 min. The injection volume was set at 5 µL. The MS was operated in negative ionization mode and the scan range set at a mass-to-charge ratio of 200-1000 Da. Data for lipid molecular species identification and quantification was obtained by product ion experiment. Data acquisition using Analyst 1.7.1 was performed with a cycle time of 560 ms with 100 ms acquisition time for MS1 scan and 10 ms acquisition time to obtain the MS2 scan of each ion. An ion spray voltage (ISVF) of −4.5 kV set to analysis. Additional parameters included curtain gas (CUR) set at 25 psi, declustering potential (DP) at 80 V, nebulizer (GS1) and heater (GS2) gases at 50 psi and interface heater of 500 °C. Collision energy (CE) for HODE ([M-H]^-^ = 295.2) and HETE ([M-H]^-^ = 319.2) were set as -30 eV and -24 eV, respectively. Specific fragments monitored for each oxidized lipid were defined as follows: 13-HODE (*m/z* 195.1391), 9-HODE (*m/z* 171.1027), 12-HETE (*m/z* 179.1078), and 11-HETE (*m/z* 167.1078). These fragments were confirmed by MS/MS experiments, comparing spectra from HODE and HETE standards with those from experimental samples (S3 Fig). The peak areas from standards and HODE and HETE isomers were obtained by using Multiquant software and a 5 mDa defined as the maximum acceptable mass error. The area ratio obtained for each oxidized lipid was calculated by dividing the peak area of the lipid by the corresponding external standard. Intensity of specific fragments of each compound was corrected by using reference standards 13-HODE, 9-HODE, 12-HETE, and 11-HETE. Data are presented in area ratio as average ± standard error of the mean.
