# Supplementary material for: Plasma lipidome profiling of newborns with antenatal exposure to Zika virus
Source: PLoS Negl Trop Dis. 2021 Apr 30;15(4):e0009388. doi: 10.1371/journal.pntd.0009388 (PMC8115770; doi:10.1371/journal.pntd.0009388)
Supplement: Attachment — Submitted filename: comments.docx [file pntd.0009388.s009.docx]

Neili and colleagues conducted plasma lipidomics analysis of newborns with and without antenatal exposure to Zika virus using LC-QTOFMS. This is an important topic and lipidomics study could shine some light on the underlining mechanism related to Zika-induced microcephaly in the newborns. The study made some interesting findings and I have the following comments:

1. The scores and loadings plots of OPLSDA were shown in Fig1. It will be good to include the scores plot of PCA as well, which can show if there’s any unsupervised grouping of the samples and QCs.
2. Fig 2A: heatmap or scatter plots may be a better way to show the changes of these lipids, which can also show the variation of the samples within each group. I would really like to see scatter plots of certain lipids across the three groups, especially those Oxy-FAs, FFAs (LA, AA, DHA etc) and those TGs linked to DHA, AA and LA, etc. This can be added as a supplementary figure. Meanwhile, FFA 15:0 and 17:0 were detected here and what could be the source of these odd-chain FFA?
3. Page 9 “statistical analysis”: why was paired t-test used? Isn’t unpaired t-test more proper here?
4. Page 10, line 194: Which figures are S1B and C Fig? I couldn’t find them.
5. The authors have pointed one of the limitations in this study was that the HODE structure (9-, or 13-HODE or both) wasn’t confirmed. This is one of the key findings in this study, so it is important to confirm the structure. In fact, 9- and 13-HODE standards are available and the two compounds can be easily separated on a c18 column. The authors can follow the LC conditions used in LCMS analysis of oxylipins and conduct a targeted analysis of 9- and 13-HODE for these samples. It can also serve as a validation of the finding from untargeted lipidomics analysis.
6. S1 Methods: lipid extraction was done by vortexing for 15s and stirring for 1h. Is it really necessary to stir for 1h? Have the authors tried to stir for short time and will it really make a difference in extraction efficacy?
