# Supplementary material for: Plasma lipidome profiling of newborns with antenatal exposure to Zika virus
Source: PLoS Negl Trop Dis. 2021 Apr 30;15(4):e0009388. doi: 10.1371/journal.pntd.0009388 (PMC8115770; doi:10.1371/journal.pntd.0009388)
Supplement: Attachment — Submitted filename: ResponseLetter_PNTD.docx [file pntd.0009388.s010.docx]

POINT-BY-POINT REBUTTAL LETTER

***********************

**Editor's comments**

The reviewers found this to be a well written and executed study, but did have some suggestions that could strengthen the manuscript. One reviewer asked for an additional experiment that seems reasonable and should be highly considered by the authors.

*R:* *We are thankful for the editor for his support in handling our manuscript. We have considered all suggestions, including the execution of an additional experiment to strengthen our case. We hope to have fulfilled all comments/issues raised by the reviewers (please see below a point-by-point response to their comments) with significant changes that considerably improved the quality of the manuscript.*

**Reviewer's Responses to Questions**

Reviewer #1: The authors investigate lipidomic profile of ZIKA congenitally infected nowborns by using high performance liquid chromatography. The method suffers from two limitations (unability to diciminate HODE isoforms and single point sampling) but they are discussed and do not critically change the manuscript message.

*R:* *We are thankful for the reviewer for accepting to review this manuscript under the circumstances that the world is facing. We agree that our manuscript has methodological limitations, and we have addressed one feasible point, from the two raised above. We have now performed an additional analysis to quantify the isomers from HODE and HETE in the samples (new* ***Fig 3****). The results are implemented into the new version and do not change the general message that HODE are increased in ZIKV-induced microcephaly relative to the other groups. However, the findings do show that 13-HODE is the most significantly altered isoform, and therefore, the new data required some arrangements in Figures, Methods, Results and Discussion. A significant amendment is revealed in the new correlation analysis (****Fig 3B****), where we used the data from total HODE (****S2 Methods****), which is more appropriated given the precise quantification by targeted oxilipidomics (see new* ***S3 Fig****). This new correlation analysis showed, in contrast to the former, a reduced number of FFA significantly correlated with total HODE concentrations. Nonetheless, it revealed that total HODE was associated with levels of several other oxidized lipids as well as to TG composed mostly of saturated and mono-unsaturated fatty acid chains. We thank the reviewer for this suggestion that considerably strengthened our conclusions, further strengthen the link between ZIKV microcephaly with oxidative and inflammatory processes. Finally, we would also like to state here that we had planned to do such experiments before submitting the manuscript to PNTD, but the Covid-19 pandemic hit us and prevented repairing of our mass spectrometry instrument on time. Basically it took over 9 months to import the parts to replace the damaged ones, and an additional 2 months to have it up and running.*

Reviewer #2: (No Response)

Reviewer #3: This study compares the plasma lipidome profiles of Zika virus exposed microcephalic and normocephalic newborns compared to uninfected controls. A key oxidized lipid, hydroxyoctadeconoic acid (HODE) derived from linoleic acid was observed to be higher in microcephalic newborns compared to the norpmocephalic group and controls. HODE was also positively associated with increased circulating free fatty acids providing a preliminary basis for a plasma lipidome signature. An additional interesting finding was the observation that lysophosphatidylcholine was higher in ZIKV exposed normocephalic newborns linking possibilities that polyunsaturated fatty acid transport may be hindered. This study is very well written and results clearly explained. The lipidomics and the statistical analyses are clearly carried out and written well in the materials and methods. Concerns regarding sample pool size is addressed in the discussion including other limitations. No concerns about ethical or regulatory requirements.

*R: We are thankful for the reviewer for the revision of our manuscript during the rough times of a worldwide pandemic. We are also thankful for the supportive comments throughout the revision. Please find below our response to your suggestion.*

--------------------

**Results**

Reviewer #1: The data sound and are presented clearly. Figures and tables are well designed and allow nice understanding of the results.

*R: We appreciate the reviewer’s support.*

Reviewer #2: (No Response)

Reviewer #3: The results are clearly explained and limitations addressed.

It would be very valuable to the readership if the authors could also provide a table of information of all the data from the untargeted analyses with included observed mass, retention time, putative IDs, accurate mass, ppm error etc so that a complete picture of the observed plasma lipidome of these newborns could be assessed with the understanding that accurate identification of the masses require further analyses.

*R: To address this comment, we implemented a new supplementary* ***S3 Table*** *including major ions, averaged retention time and exact masses of all lipids reported and their respective internal standards. It must be emphasized that our untargeted approach is based exclusively on MS/MS fragmentation patterns (emphasized in* ***S1 Methods****), from which a list of ions and their retention times is created. These ions are then quantified by their MS spectra using a 5 mDa limit from their exact mass values. We have also now included a statement in the* ***S1 Methods*** *section so that upon reasonable request, the corresponding author will provide any further detail on the MS analysis, including sharing the raw data.*

--------------------

**Conclusions**

Reviewer #1: (No Response)

Reviewer #2: (No Response)

Reviewer #3: The conclusions based on this limited dataset are valid and are well discussed compared to the current literature.

*R: We appreciate the reviewer’s support.*

--------------------

**Editorial and Data Presentation Modifications**

Reviewer #1: There is a reference displayed as "Kikut et al., 2020", line 281, unlike the others, displayed as numbers.

*R: We thank the reviewer for this remark, and we have now corrected this typo.*

Reviewer #2: (No Response)

Reviewer #3: (No Response)

--------------------

**Summary and General Comments**

Reviewer #1: Viral infection during pregnacy may associate with alteration of lipid metabolism, thereby threatening homeostasis and development. Here the authors show that the lipidomic profile of newborns congenitally infected by ZIKV differs from that of control subjects and show increased HODE levels. The findings provide new insight on ZIKV infection pathophysiology. They disclose new clues to understand neurological sequelae since elevated HODE levels have been already associated with HCMV infection, even though no causality can be formally established.

*R: We appreciate the reviewer’s general support of our manuscript and agree that future studies on the causality role of oxidized fatty acids on ZIKV neuropathogenesis are warranted. To account for this comment we now state: "* *Secondly, quantification of HODE isomers revealed that concentrations of 13-HODE, and to a lesser extent 9-HODE, are elevated in plasma from newborns with ZIKV-induced microcephaly. While a direct role of 9-HODE impairing neurogenesis in stem cells infected by cytomegalovirus has been established [58], a causal link between HODE isomers and the neuropathogenesis of ZIKV remains to be investigated."*

Reviewer #2: (No Response)

Reviewer #3: While this is a limited data set and limited number of samples that are challenging to acquire, the information resulting from this study are clearly presented and can drive future analyses of how lipids might control newborn brain development as well as how ZIKV might alter this process.

*R: We appreciate the reviewer’s supportive comments that reporting initial insights on cord plasma lipidome may contribute to future studies investigating ZIKV-induced pathophysiology in newborns.*

--------------------

**Additional Comments**

Neili and colleagues conducted plasma lipidomics analysis of newborns with and without antenatal exposure to Zika virus using LC-QTOFMS. This is an important topic and lipidomics study could shine some light on the underlining mechanism related to Zika-induced microcephaly in the newborns. The study made some interesting findings and I have the following comments:

1. The scores and loadings plots of OPLSDA were shown in Fig1. It will be good to include the scores plot of PCA as well, which can show if there’s any unsupervised grouping of the samples and QCs.

*R: We have used QC samples, intentionally injected after a batch of experimental samples, to calculate the coefficient of variance (CV) for each compound. Instead using a PCA, we have set a cut-off of <20% for CV, thereby eliminating 13 lipids prior to any statistical analyses. The purpose of using QC samples was to constrain our statistics to lipids that displayed relatively low fluctuations in concentrations throughout the measurements by LC-MS. Nonetheless, we have attached a PCA score plot below (and respectfully decided to leave it out of the manuscript) that clearly show QC samples segregated from experimental samples.*

2a. Fig 2A: heatmap or scatter plots may be a better way to show the changes of these lipids, which can also show the variation of the samples within each group. I would really like to see scatter plots of certain lipids across the three groups, especially those Oxy-FAs, FFAs (LA, AA, DHA etc) and those TGs linked to DHA, AA and LA, etc. This can be added as a supplementary figure.

*R: We considered using a heatmap for displaying the data, but since we conducted pairwise contrasts, it could mislead interpretations on the magnitude of alterations in specific comparisons. In retrospect, we agree with the reviewer's suggestion that scatter plots of altered lipids across the 3 groups, especially those modulated in two contrasts could be helpful for data interpretation. To account for this suggestion, we implemented a new supplementary* ***S2 Fig*** *displaying scatter plots of the concentrations of selected lipids, focusing on the significantly altered ones (including many FFA). Concerning the concentrations of polyunsaturated fatty acids, we have implemented* ***Fig 3A*** *for the major Oxy-FA and have shown in* ***S1 Fig*** *the distribution of fatty acids in TG. We suspect that* ***S1 Fig*** *was not attached to the former version of the manuscript for revision, and we will make sure it will be included in this revised version.*

2b. Meanwhile, FFA 15:0 and 17:0 were detected here and what could be the source of these odd-chain FFA?

*R: The occurrence of odd-chain fatty acids in the human plasma has been a topic of several excellent reviews (e.g. Jenkins et al. Molecules (2015) 20(2): 2425–2444). The potential sources of odd-chain fatty acids have been linked to (but not exclusively to) dairy products, gut microbiota and endogenous production. Regarding the latter, interesting possible pathways of synthesis have been proposed (please see Jenkins et al., 2015; Huynh et al., 2019, Cell Chemical Biology 26, 71–84). However, because these potential sources are not fully constrained, we think that including such discussion would detract the reader from the fact that increased concentrations of FFA, both odd- and even-chain, could lead to lipotoxicity of cells/tissues.*

3. Page 9 “statistical analysis”: why was paired t-test used? Isn’t unpaired t-test more proper here?

*R: We thank the reviewer for this very important remark. This was a typo that we have now corrected throughout the main text to read: "unpaired t-test"*

4. Page 10, line 194: Which figures are S1B and C Fig? I couldn’t find them.

*R: These data were shown in supplementary* ***S1 Fig*** *of the submitted version. We suspect that the revision document did not contain this information, but we will ensure that it will be available this time.*

5. The authors have pointed one of the limitations in this study was that the HODE structure (9-, or 13-HODE or both) wasn’t confirmed. This is one of the key findings in this study, so it is important to confirm the structure. In fact, 9- and 13-HODE standards are available and the two compounds can be easily separated on a c18 column. The authors can follow the LC conditions used in LCMS analysis of oxylipins and conduct a targeted analysis of 9- and 13-HODE for these samples. It can also serve as a validation of the finding from untargeted lipidomics analysis.

*R: We have now implemented the analysis of HODE and HETE isoforms by a targeted oxilipidomic approach. This analysis allowed confirming the structures based on their MS/MS fragments (please see the implemented S3 Fig and* ***S2 Methods****). The implementation of this analysis enabled not only validating our former results from the untargeted analysis, but also revealed that 13-HODE concentrations are about one order of magnitude higher than 9-HODE in these samples. These and other aspects relative to the new analysis of HODE and HETE isomers are now detailed in the main text, figure, and methods. We again thank the reviewer for this suggestion, which substantially improved the manuscript.*

6. S1 Methods: lipid extraction was done by vortexing for 15s and stirring for 1h. Is it really necessary to stir for 1h? Have the authors tried to stir for short time and will it really make a difference in extraction efficacy?

*R: This is an interesting point to be verified for optimization of lipid extraction. In this study, however, we have followed the procedures by Matyash et al (2008; ref 41), without altering the stirring time. Some 5 years ago, our laboratory tested several conditions before applying the method. Although not formally published, we noticed a decrease in yields and an increase in data variability by shortening the stirring time to 30 min. For this reason, since then we apply the original protocol to our samples.*
